# Discovering and comparing types of general practitioner practices using geolocational features and prescribing behaviours by means of K-means clustering

**DOI:** 10.1038/s41598-021-97716-3

**Published:** 2021-09-14

**Authors:** Frederick G. Booth, Raymond R Bond, Maurice D Mulvenna, Brian Cleland, Kieran McGlade, Debbie Rankin, Jonathan Wallace, Michaela Black

**Affiliations:** 1 School of Computing, Ulster University Jordanstown, Belfast, Northern Ireland UK; 2 Dunluce Family Practice, Belfast, UK; 3 School of Computing, Engineering and Intelligent Systems, Ulster University, Magee Campus, Londonderry, Northern Ireland UK

**Keywords:** Statistics, Computer science

## Abstract

Traditionally General Practitioner (GP) practices have been labelled as being in Rural, Urban or Semi-Rural areas with no statistical method of identifying which practices fall into each category. The main aim of this study is to investigate whether location and other characteristics can provide a tautology to identify different types of GP practice and compare the prescribing behaviours associated with the different practice types. To achieve this monthly open source prescription data were analysed by practice considering location, practice size, population density and deprivation rankings. One year’s data was subjected to k-means clustering with the results showing that only two different types of GP practice can be classified that are dependent on location characteristics in Northern Ireland. Traditional labels did not describe the two classifications fully and new classifications of Metropolitan and Non-Metropolitan were used. Whilst prescribing patterns were generally similar, it was found that Metropolitan practices generally had higher prescribing rates than Non-Metropolitan practices. Examining prescribing behaviours in accordance with British National Formulary (BNF) categories (known as chapters) showed that Chapter 4 (Central Nervous System) was responsible for most of the difference in prescribing levels. Within Chapter 4 higher prescribing levels were attributable to Analgesic and Antidepressant prescribing. The clusters were finally examined regarding the level of deprivation experienced in the area in which the practice was located. This showed that the Metropolitan cluster, having higher prescription rates, also had a higher proportion of practices located in highly deprived areas making deprivation a contributing factor.

## Introduction

As large datasets have become increasingly available, academics and healthcare researchers have sought to create meaningful visualisations to help understand trends hidden within the data^1^. Some studies have aimed at presenting this information geographically showing trends at county level in the United States^2^ whilst others attempt to create new ways of visualising geolocational time series data^3^, but it is generally accepted that geographical references within medical data have not been adequately explored^4^. Whilst the majority of studies utilise ‘Big Data’ provided for specific studies, a wealth of ‘Open Data’ has now become available for analysis. The standardisation of the data being published^5^ has meant that different data sets can now be linked, enhancing the wealth of information contained within them. Recently, Open Data has been utilised to investigate the links between antidepressants, deprivation, and suicide in Northern Ireland and to identify anomalies^6^. This study used ‘open’ prescription data to identify levels of antidepressant use in Northern Ireland. Elsewhere, prescription data has been used to analyse prescription patterns in hospitals^7^, prescription patterns while taking into account the patient’s symptoms^8^, to track disease and examine seasonality^9^ and gain insights into the differences in prescription behaviour of individual GP practices^10^. Whilst other studies have examined the differences in prescribing behaviours in general^11^ and for specific medications such as antidepressants^6^ and hormone therapy^12^, no studies have been identified examining prescribing behaviours a method of classifying GP practices.

It has generally been accepted by healthcare researchers that General Practices are in Urban, Rural and Semi-Rural areas^13^ although many studies focus on the Rural / Urban split as a binary division^14^ ignoring semi-rural practices. The few studies addressing healthcare in semi-rural areas class these areas as being “a small town, and the surrounding rural area”^15^ but do not present any formal mechanism for this classification. On further investigation, formal mechanisms do exist for the classification of geographical areas based on population^16,17^, remoteness of the area^18^ and even the provision of services in the area^19^. Yet no previous studies have been identified proposing a formal method for classifying GP practices in terms of geographical location and their relationship with the pharmacies that dispense their prescriptions. This study will test the hypothesis that location is an important feature and that General Practices can be classified in terms of characteristics/features pertinent to their location and associated pharmacies. The objectives of this study are to discover how clustering techniques, utilising the geographical features associated with GP practices, can identify practice types and to compare the prescription rates between the identified clusters over British National Formulary (BNF) chapters^20^.

### Aims and objectives: research questions

No previous studies have been identified that seek to classify General Practices in terms of geographical location and associated attributes. In fact, no statistical method has been identified for the categorisation of GP practices. This means that different studies may not be comparing like for like resulting in different outcomes and conclusions. It is vital that future classification of GP practices use a standard method, and this study aims to examine the importance of location and prescribing profile in this classification process. By providing a standard statistical method of classification, the structure and prescribing profiles of practices can be compared globally. To make this process as simple as possible, geolocation and prescribing based features have been subjected to k-means clustering. This study also aims to investigate the prescribing behaviours of identified clusters of practices to determine the effects of geolocation and to elucidate whether it is possible to:

- classify GP practices using their prescription profile
- make meaningful distinctions between practices using practice size, prescribing profile and geolocation of practices and pharmacies
- identify clusters of practices which differ in their prescribing patterns
- attribute differences in prescribing behaviours to location alone or, if not, identify other contributing factors.

## Methods

### Data sources

This analysis has been performed using two main data sources, ‘Dispensing by Contractor’^21^ and ‘GP Prescribing in Northern Ireland’^22^ datasets provided by the Health and Social Care Business Services Organisation via the Open Data NI Portal. The ‘Dispensing by Contractor’ dataset first became available from April 2018, and hence analysis was limited to the 1-year period from April 2018 to March 2019. In addition, the data was enhanced using the Postcode to Super Output Area (SOA)^23^ and Postcode to Ward^24^ lookup tables published by the Office for National Statistics (ONS), Population Density figures for SOA’s^25^ and Deprivation Rankings by Ward^26^ from the Northern Ireland Statistics and Research Agency (NISRA), and GP Practice size figures^27^ provided by the Department of Health Business Services Organisation that were also available on the Open Data NI portal.

**Figure 1:**
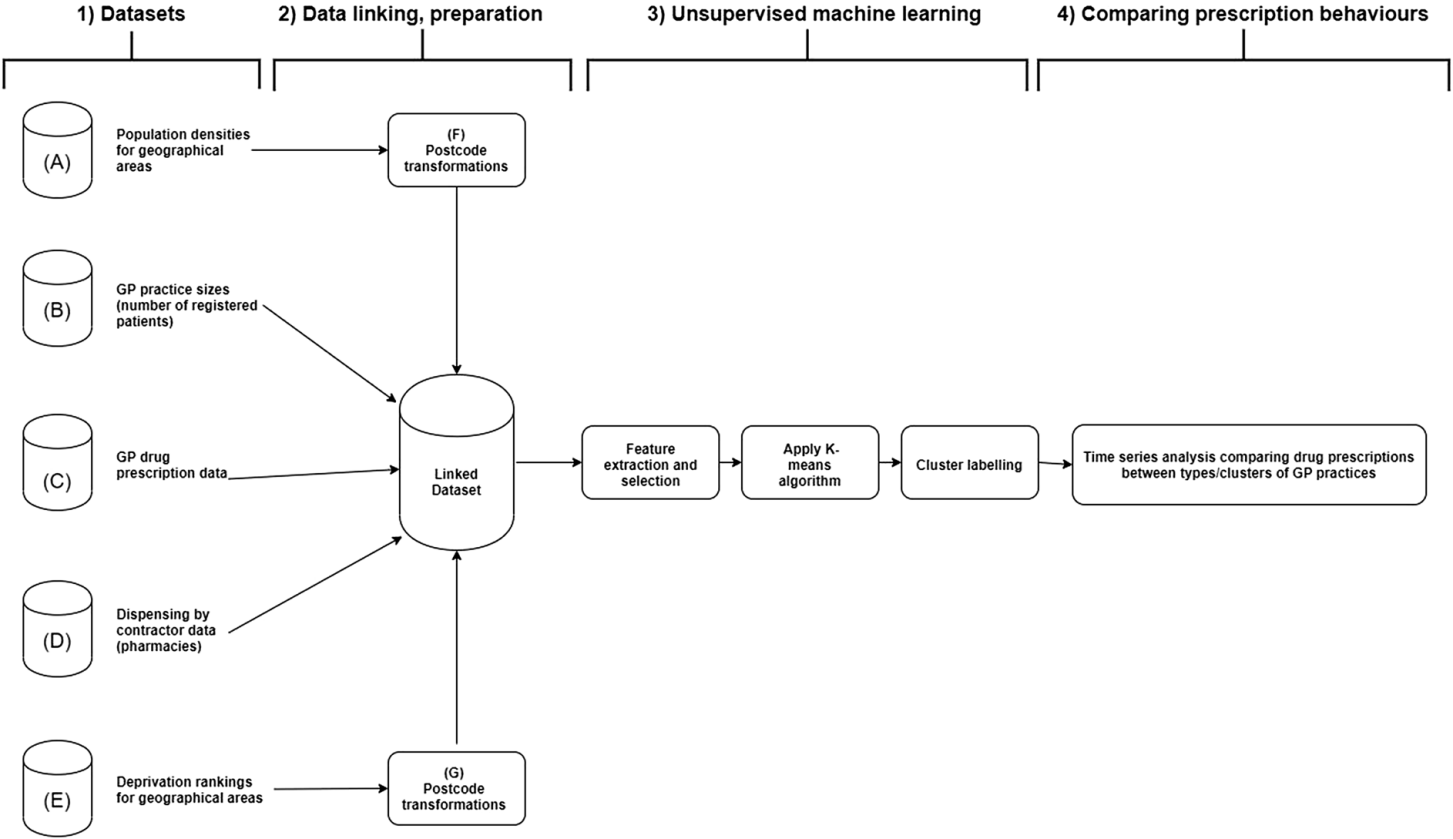
Workflow used for investigating prescription behaviours of General Practitioner practices based on Geolocation using Time Series Analysis and K-means Clustering.

Figure 1 shows the datasets used in this analysis with the associated workflow. The ‘Dispensing by Contractor’ dataset (D) provided postcode information on both the practice issuing the prescription and the pharmacy dispensing it along with the number of items prescribed. Each monthly dataset consisted of approximately 18,400 records with 15 variables making the combined dataset for April 2018–December 2019 almost 221,000 records in length. The purpose of this analysis was to examine GP practices using geolocation as the main feature in describing each practice. As part of this it was important to include the geographic sphere of influence of each practice, and, as no geographical data on patients was available, the pharmacies used to dispense prescriptions issued by practices were used as a proxy. Postcodes were converted into Northings and Eastings using an online geocoding service^28^ and these were used to calculate the distance between practice and pharmacy used in the formula: the quotient being the square root of the difference in northings squared plus the difference in eastings squared divided by one thousand. Postcodes were also used to link each practice to the population density of the area in which it was located using the Postcode to Super Output Area (SOA) table (F) provided by the and Deprivation Rankings by Ward using the postcode to ward conversion table (G) from ONS. Practice Size data (B) is published every three months provided the number of registered patients in each GP practice with the same figure being applied to each practice for the month of release and the following two months.

### Clustering

From the available data six features were chosen which best represented the relationship of a practice with its geolocation and prescribing profile. As geographical coordinates are not linear and pose a challenge for machine learning, distance was used as a proxy for geolocation resulting in the following features. The median distance travelled along with the associated standard deviation formed the backbone of the key features describing GP practices. The standard deviation of distance travelled shows the variability of the distances travelled. The other features identified include the number of pharmacies who service each practice along with the number of items dispensed per registered patient per month, the number of registered patients in the practice and the population density of the SOA in which the practice is located. These features were then used to perform clustering analysis on the dataset (Table 1).

**Table 1:** Summary of Features used for clustering.

Feature	Description	Rationale
Number of Pharmacies	The number of pharmacies dispensing prescriptions issued by a practice	This is fundamental to the operation of any practice and helps define the area in which the practice operates
Number of Items per Registered Patient	The number of items prescribed by a practice and subsequently dispensed by a pharmacy. This figure is normalised by dividing by the number of registered patients in the practice	The level of prescribing is used as a proxy for the levels of sickness experienced by registered patients in the practice
Median Distance to Pharmacy (km)	The distance travelled to a pharmacy to dispense a prescription	This provides an insight into patients’ behaviours and can be used as a proxy for the average distance patients live/work from the practice
Distance Standard Deviation (km)	The standard deviation of the distance travelled to a pharmacy	This variable shows the extent to which the average distance may vary
Population Density per Square km	The population density of the area in which the practice is located	This gives an indication of the number of people living in the area in which the practice is located and relates to the rural/urban character of the area
Registered Patients	The number of registered patients in the practice	This gives us an indication of the size of the practice

In preparation for clustering, the data for each practice was collated to provide a single datapoint for each feature. The distribution graph for distance showed that this variable was skewed to the left and therefore not Gaussian. For this reason, the median for this feature was used for clustering. Over a twelve-month period, the pharmacies where a particular GP practice prescription is dispensed are counted uniquely.

Unsupervised clustering was performed on the data with the aim of identifying patterns or clusters within the data. To achieve this, it was important to choose the most relevant features to cluster on. Important features help to create clusters and unimportant features may hinder the formation of clusters. As the various features have different scales, it was important to normalise the dataset to improve the accuracy of the clustering algorithm. There are several clustering algorithms available, this study used the k-means algorithm to group data points together based on their Euclidean distance from each other^1^. K-means was chosen for its simplicity in both parameters to be set (k and the number of times to run the algorithm) and its propensity for not being memory intensive allowing other researchers to easily follow the same method. Clusters were observed and using centroid data and geographical visualisation labels were assigned to each cluster and applied to the original dataset.

The dispersion of pharmacies (distance between pharmacies dispensing prescriptions for a given practice) for each cluster type was examined to see if this information would support the cluster labelling established in the previous step. To do this the average distance between each pharmacy dispensing prescriptions for a particular practice was calculated.

### Practice prescribing

The prescription trends for identified clusters were identified by linking the practices to the monthly breakdown of prescriptions provided by the ‘GP Prescribing in Northern Ireland’ datasets (D). These monthly datasets provided a breakdown of prescriptions by practice, by BNF chapter and section. Each monthly dataset comprised approximately 456,600 records with 16 variables, with the complete dataset (July 2015–December 2019) having over 24,600,000 records. This dataset was then cleaned with records not having BNF Chapter or Section information being assigned as chapter 99 and excluded from any further analysis. These comprised approximately 0.24% of the total records.

In order to ensure that ‘like for like’ comparisons were made, “the total items prescribed” data was normalised using the registered patient’s data for each practice, resulting in a “prescriptions per patient” figure.

Root Mean Square Error (RMSE) was used to compare the differences between the month-by-month time series prescribing patterns of two identified clusters. RMSE is a frequently used metric to measure the differences between predicted values and those observed but can also be used to measure the difference between any two sets of values. This is done by calculating the distance between data points, known as the residual, and the square root of the mean of the squares of all residuals being computed.

The Python programming language and Jupyter Notebooks were used throughout, utilising Pandas DataFrames for data wrangling and Scikit-Learn (Version 0.21.2) for clustering. Matplotlib^29^ and IpyLeaflet^30^ were used for data visualisation.

### Deprivation

Having identified clusters of GP practices, their prescribing patterns were examined at BNF chapter level to identify any differences. Chapters showing significant differences in prescribing behaviour were then examined at Section level to try to pinpoint the reason for these differences. Finally, each cluster of practices was examined considering the Deprivation ranking of the area in which each practice was located in order to ascertain whether deprivation was a contributing factor to the identified trends in prescribing behaviour.

## Results

Initial analysis identified 333 practices operating during the 12 months from April 2018 to March 2019 and showed that although the majority of prescriptions generated by a particular practice were dispensed by a pharmacy within the local area, a significant number were also dispensed at distances up to 100 km away.

Using the six features identified to describe a practice (Table 1), k-means clustering was used to identify different types of practice. The elbow method was used to identify the optimum number of clusters. The expected elbow in the graph indicated that the optimum number of clusters was two and this was corroborated using the Silhouette method (Appendix 1).

**Figure 2:**
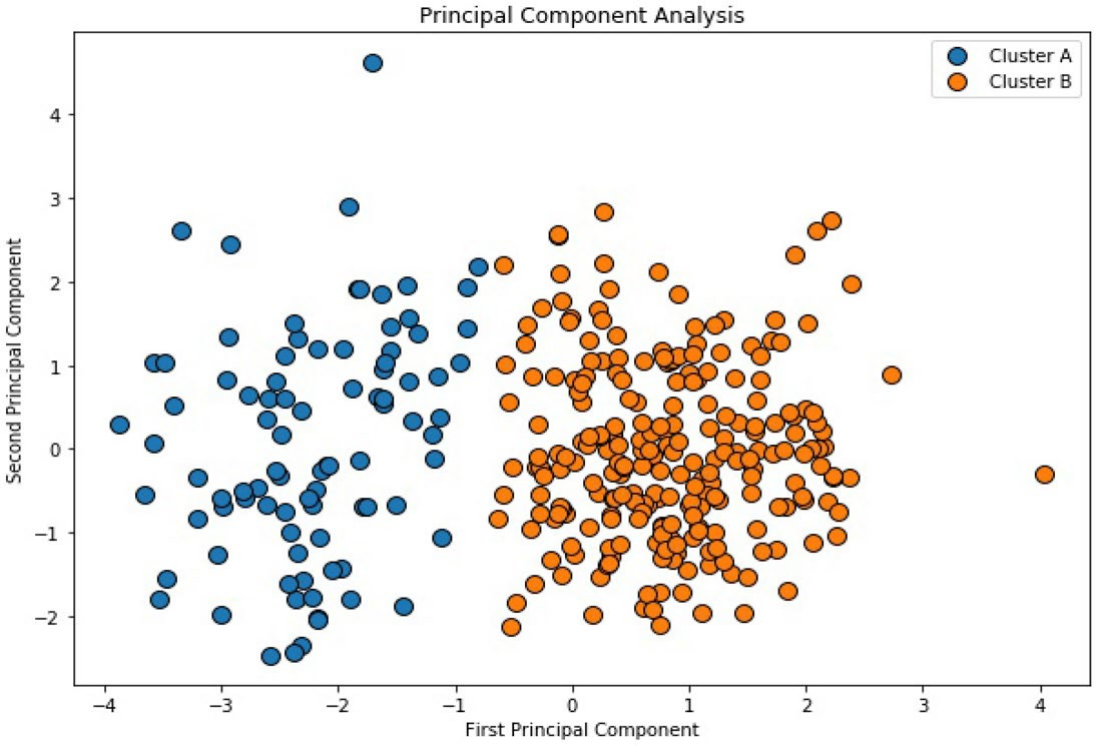
Principal Component Analysis (PCA) Plot.

**Figure 3:**
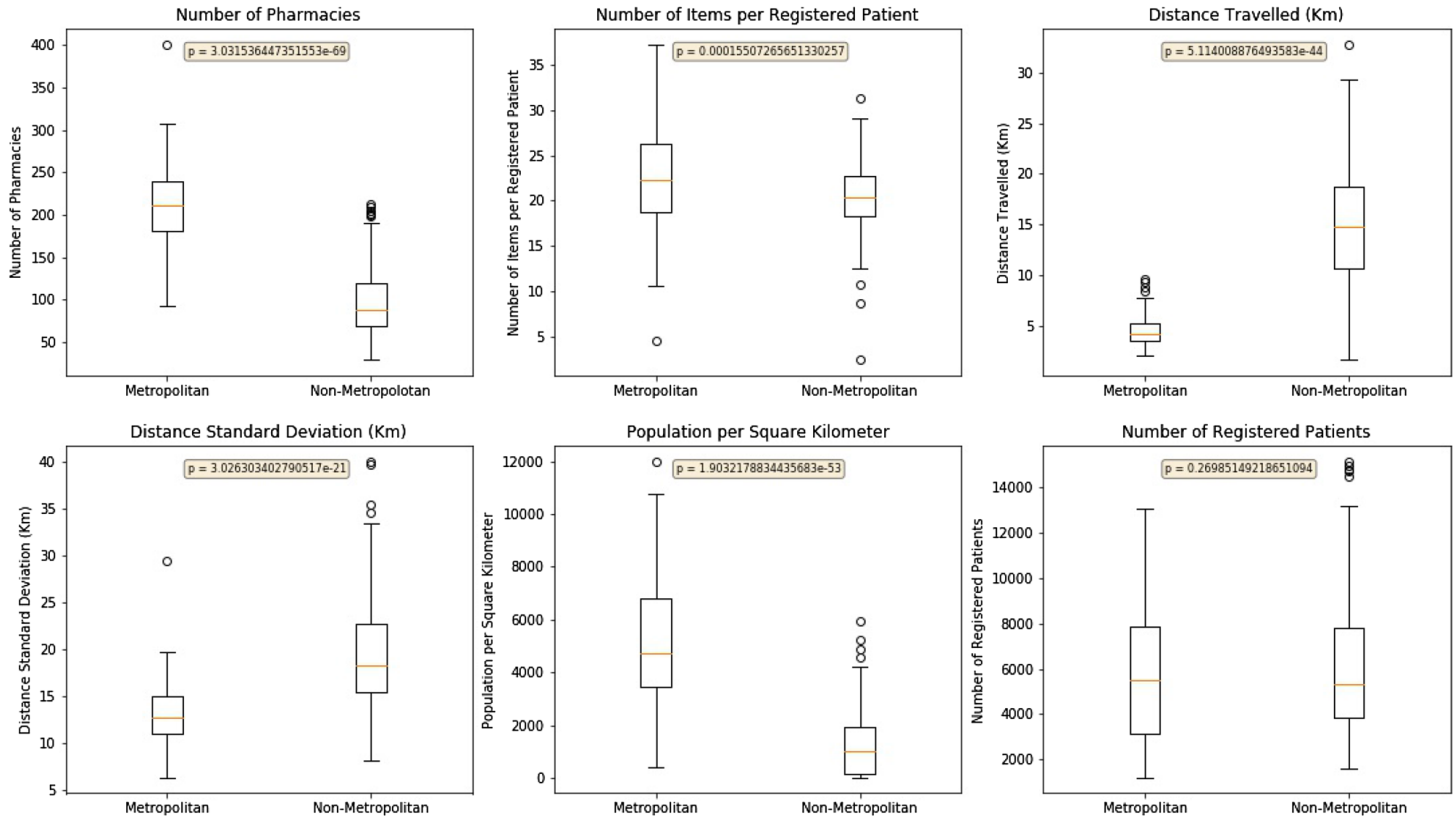
Feature Boxplots.

**Figure 4:**
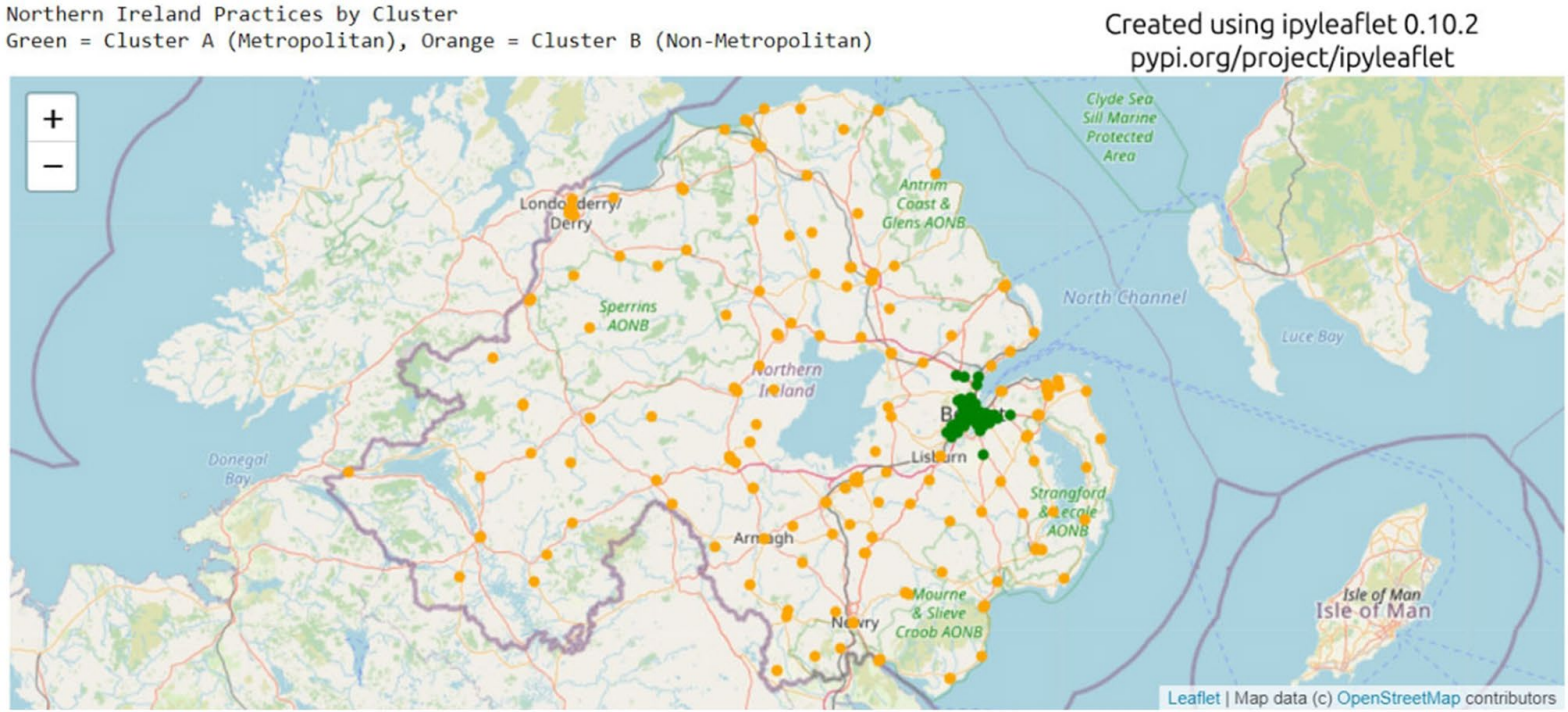
Map of GP practices by Cluster in Northern Ireland (grey line denotes the border of Northern Ireland).

Principal Component Analysis (PCA) was used to convert the six identified features of each practice into a two-dimensional array of uncorrelated variables for visualisation. The resulting graph (Fig. 2) shows two distinct clusters. Identified clusters were mapped back to the original dataset and their associated metrics examined using boxplots to show the mean, interquartile range, and outliers (Fig. 3). It is notable that the number of registered patients does not contribute significantly to the difference in the two clusters and could be ignored in future calculations. Centroid data was also calculated for each identified cluster (Table 2). These were then used as a basis to characterise the practice archetypes and to name each cluster. From the observations and the geographical locations of practices in cluster A, we classified this cluster with the label ‘Metropolitan’ given that it had a high number of pharmacies serving an area with a high population density located in the largest city in Northern Ireland. The lower number of items prescribed, and the shorter distances travelled also support this. Similarly, from the observations and the geographical locations of practices in cluster B, we originally surmised that with the longer distances being travelled and the lowest population density this cluster should be classified as Non-Metropolitan as these practices are located in both areas commonly regarded as rural and the other cities within Northern Ireland (Fig. 4).

**Table 2:** Clusters identified and shown geographically in Fig. 4.

	Metropolitan practices (Cluster A)	Non-Metropolitan practices (Cluster B)
Number of practices	90 (27%)	243 (73%)

Metropolitan practices account for 27% of the total practices in Northern Ireland with the majority of practices (73%) being classed as Non-Metropolitan (Table 2).

As can be seen in Table 3, a typical Metropolitan practice is one that normally has over 200 pharmacies associated with it, typically prescribing around 269 items per patient per year. These patients usually travel almost 5 km to collect their medication but could travel up to 18 km. These practices are typically located in areas of high population density with over 5,000 people per square kilometre and have around 5600 registered patients. A typical Non-Metropolitan practice is one that normally has under 100 pharmacies associated with it, typically prescribing around 246 items per patient per year. These patients usually travel almost 15 km to collect their medication but could travel up to 35 km. These practices are typically located in areas of lower population density with around 1,300 people per square kilometre and have around 6,000 registered patients.

**Table 3:** Archetypical characteristics each cluster (i.e. Centroid Feature Values) for the period April 2018–March 2019.

	Archetypical Metropolitan practice (Cluster A)	Archetypical Non-Metropolitan practice (Cluster B)
Number of Pharmacies	212 (+ −46.8)	98 (+ −38.4)
Number of Items per Registered Patient	268.8 (+ −5.8)	246 (+ −3.4)
Distance to Pharmacy (km)	4.6 (+ −1.5)	14.8 (+ −5.9)
Distance Standard Deviation (km)	13.1 (+ −3.4)	19.8 (+ −5.9)
Population Density per Square km	5180 (+ −2578)	1272 (+ −1230)
Registered Patients	5645 (+ −2724)	6030 (+ −2859)

**Figure 5:**
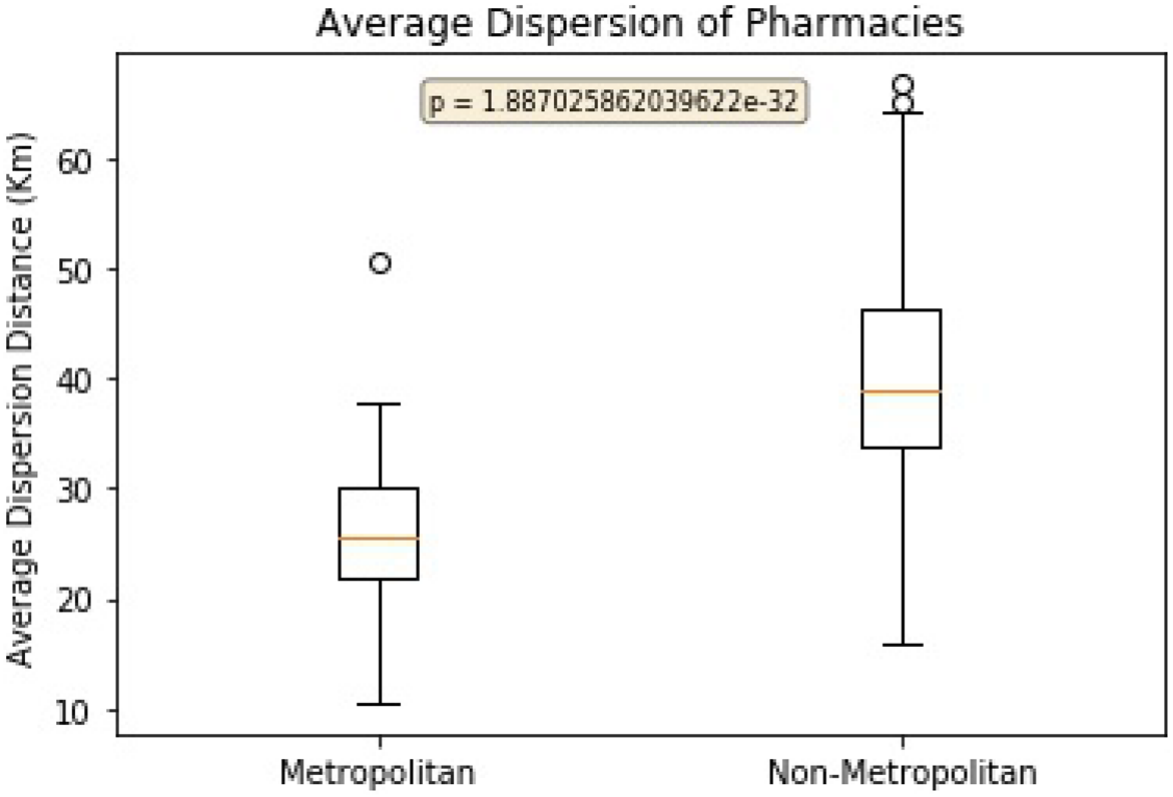
Dispersion of Pharmacies.

Calculating the average distance between pharmacies dispensing prescriptions for each practice produced an average dispersion distance measured in kilometres (Fig. 5). These figures supported the cluster labelling with Metropolitan pharmacies on average 26.2 km apart with a standard deviation of 6.3 km and Non-Metropolitan pharmacies on average 40.4 km apart with a standard deviation of 9.6 km.

### BNF chapter analysis

**Figure 6:**
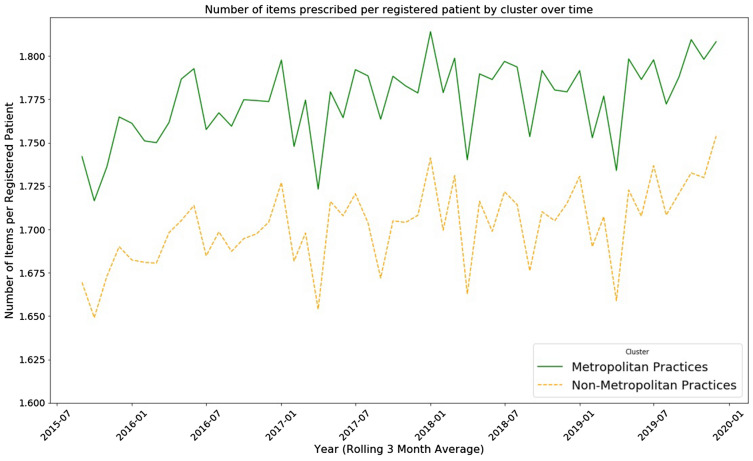
Number of items prescribed per registered patient by cluster over time.

The total number of items prescribed per patient were analysed by cluster for the period July 2015 to December 2019, the period in which all 333 practices were operational. Overall figures showed that there were similar trends for both clusters with Metropolitan practices having the highest prescription rates and Non-Metropolitan practices the lowest (Fig. 6).

**Figure 7:**
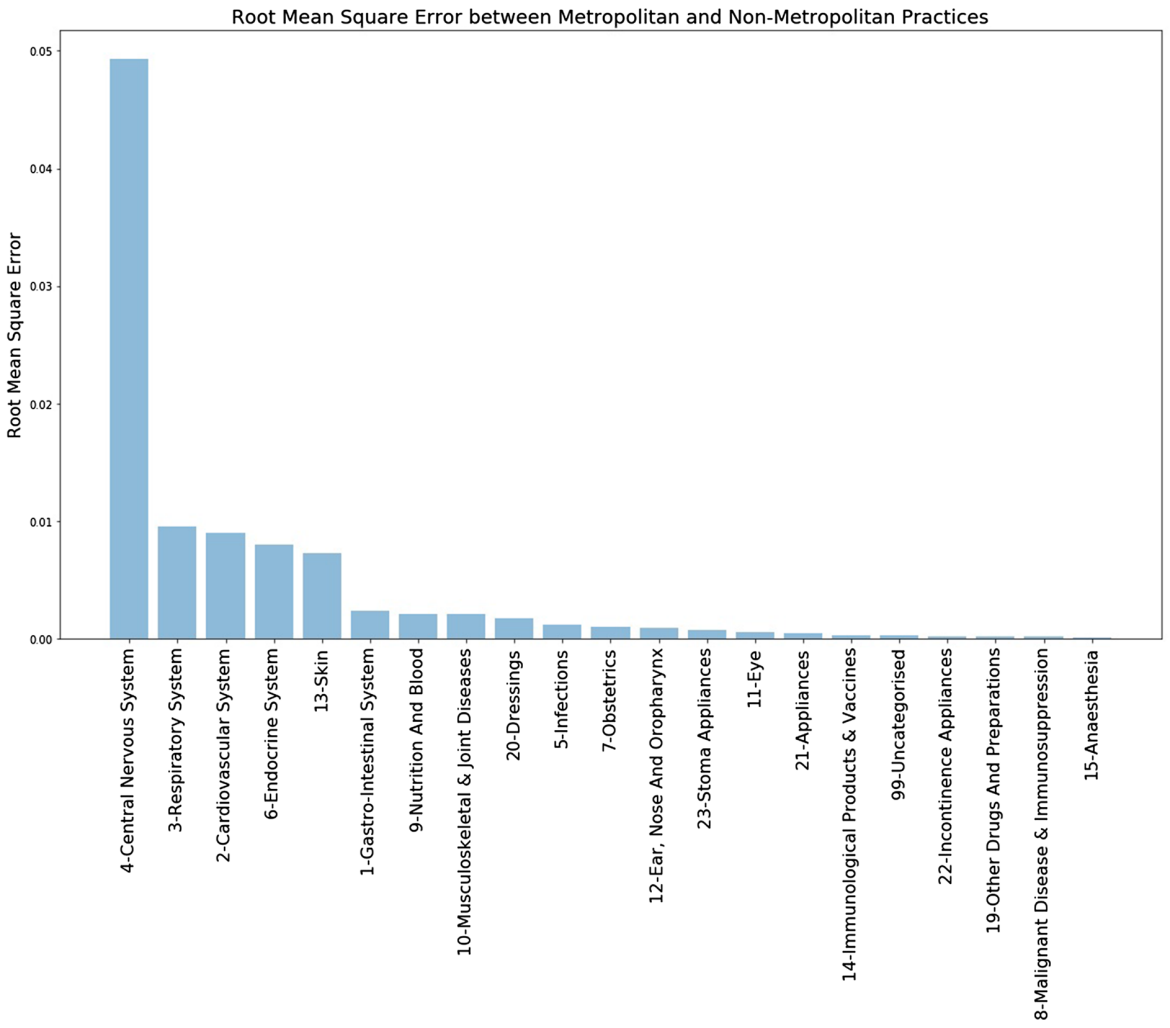
Comparison of RMSE by cluster at BNF chapter level.

Investigating this further, the figures were broken down into BNF chapters for comparison. It was found that Metropolitan prescribing was only higher in approximately half of the BNF chapters (Appendix 2). RMSE was calculated between the clusters (Fig. 7). This showed that the largest variation between clusters occurred in chapter 4 (Central Nervous System) with smaller variations evident in chapters 3 (Respiratory System), 2 (Cardiovascular System), 6 (Endocrine System) and 13 (Skin). These, and the remaining chapters, although interesting, did not contribute greatly to the overall variations observed.

### BNF Chapter 4—Central nervous system

**Figure 8:**
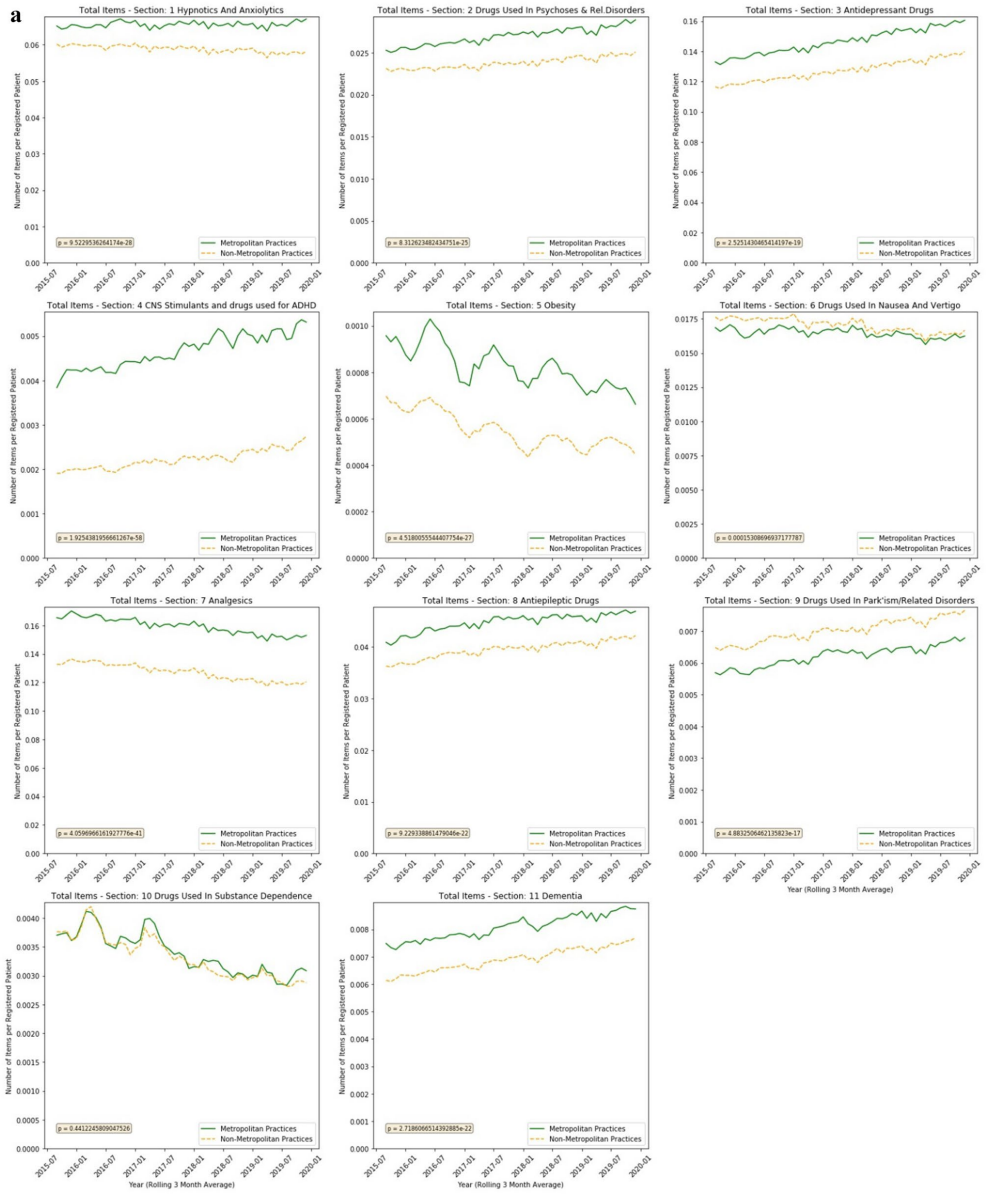

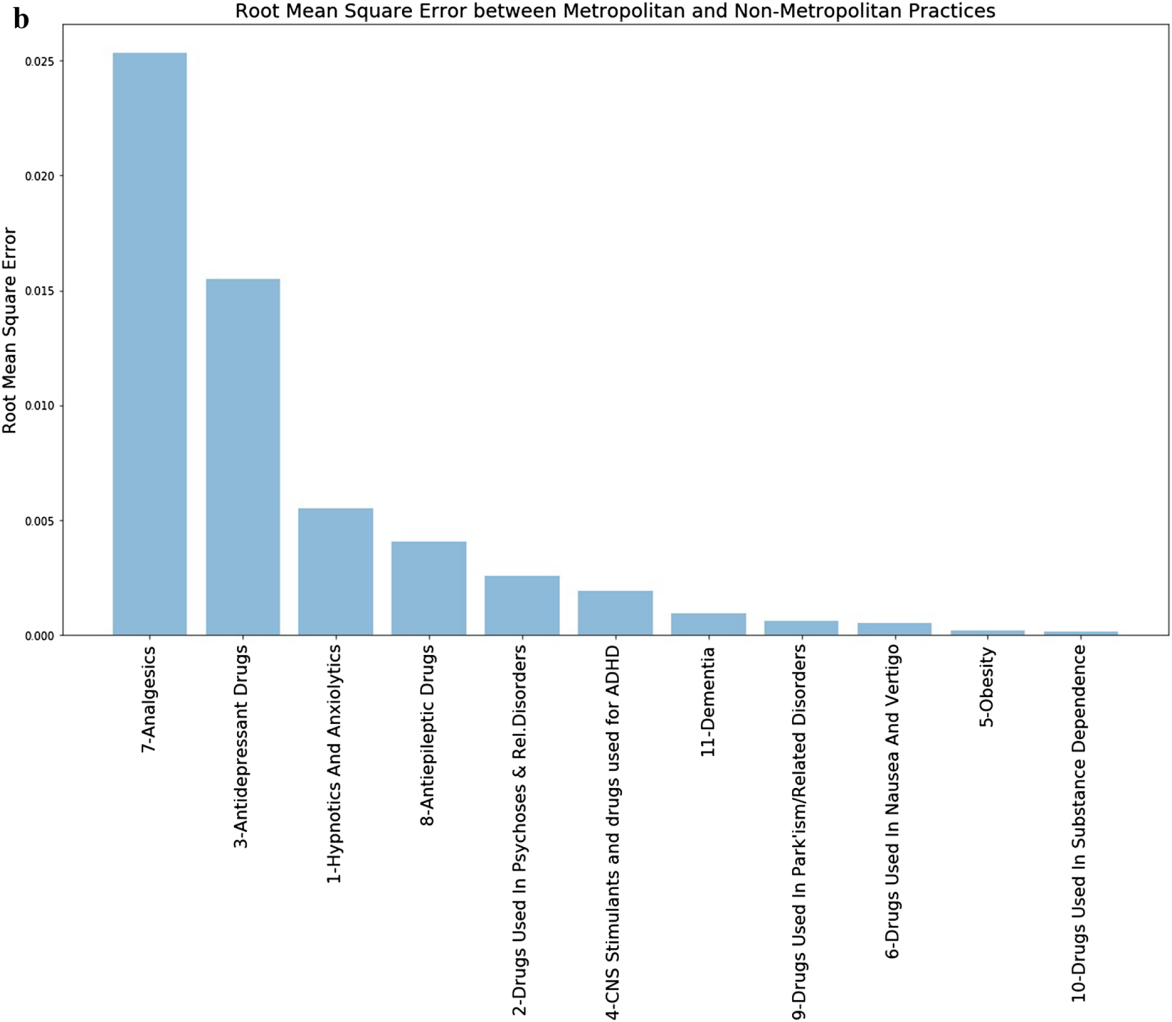
(**a**) Items per Patient for BNF Chapter 4 (Central Nervous System) Sections. (**b**) Comparison of RMSE by cluster at BNF section level (Chapter 4).

Having the highest contribution to the overall variations, chapter 4 was examined at section level (Fig. 8a) showing higher prescribing levels in nine of the eleven sections in Metropolitan practices. Only in sections 6 (Drugs used in nausea and vertigo) and 9 (Drugs Used in Parkinsonism/Related Disorders) were prescribing levels higher in Non-Metropolitan practices.

Performing independent t-tests on the sections revealed that statistically significant differences existed in all sections except for section 10 (Drugs Used in Substance Dependence).

The main contributors to the variation seen in this chapter come primarily from section 7 (Analgesics) and secondly from 3 (Antidepressant Drugs) (Fig. 8b) showing that prescribing in these two sections are considerably higher in Metropolitan practices than in Non-Metropolitan practices.

To understand whether location is a contributor to the differences in prescription rates between clusters, each practice was examined with respect to the level of deprivation assigned to the Ward in which it was located. Each cluster of practices was then summarised showing the number of practices in the first quartile (those in the least deprived Wards) up to the fourth quartile (those in the most deprived Wards) (Table 4).

**Table 4:** Percentage of Practices in Deprived Areas (Q = Quartile).

Cluster Label	Q1 (%)	Q2 (%)	Q3 (%)	Q4 (%)
Metropolitan	23.3	4.4	20.0	52.2
Non-Metropolitan	25.9	32.5	27.8	13.9
All NI practices	25.2	24.9	25.5	24.3

This analysis shows that 72.2% of Metropolitan practices are in the top 2 quartiles whilst only 50.7% of Non-Metropolitan practices would fall into the same categories. The higher levels of deprivation seen in Metropolitan practices may explain the higher prescribing levels than those of Non-Metropolitan practices.

## Summary of results

Using geolocation as the main feature in describing a GP practice has provided insights into the types of General Practitioner practices that exist nationwide. This work has indicated there are two types of practice, classified as Metropolitan and Non-Metropolitan. From the perspective of the data used in this analysis, there is no evidence for the Semi-Rural classification commonly used by healthcare researchers. Comparing prescription trends of these clusters showed that prescription rates were generally higher in the Metropolitan cluster and lower in the Non-Metropolitan cluster. Examining the prescription levels over time at BNF chapter level showed that one chapter contributed the highest proportion of variation between the cluster types, namely chapter 4 (Central Nervous System). Smaller variations were observed in chapters 3 (Respiratory System), 2 (Cardiovascular System) 6 (Endocrine System) and 9 (Skin). Examining the chapter contributing the highest variation between clusters, chapter 4 (Central Nervous System), it was found that the variation was primarily due to variations in section 7 (Analgesics) and secondly, variations in section 3 (Antidepressant Drugs). Whilst the other BNF chapters were interesting in themselves, they did not contribute much to the overall variations observed. Analysis of practices with respect to the level of deprivation experienced in their location showed that a higher proportion of Metropolitan practices are located in high deprivation areas than those in Non-Metropolitan areas.

## Discussion

In trying to establish whether GP practices could be categorised using geographical features as the basis for categorisation, two categories (clusters) have been discovered. Traditionally, the categories Urban and Rural have been used in medical circles but a formal means of identifying which practices belonged to which category did not exist previously other than their geographical location.. No evidence has been found supporting a third, Semi-Rural classification in Northern Ireland commonly used by clinicians. Rural and Urban have been used as binary indicators in previous research^14^ with Semi-Rural being loosely defined as “a small town, and the surrounding Rural area”^15^ but no other justification for this classification is given. It was found that these labels did not fully describe the categories found in Northern Ireland, hence Metropolitan and Non-Metropolitan were used instead. From the total items prescribed by the cluster graph (Fig. 5) it can be clearly seen that the two cluster types have different prescribing levels with Metropolitan consistently being the highest over the period July 2015 to December 2019, and Non-Metropolitan being the lowest. It is also evident that both clusters are highly correlated and have similar trends over time.

Breaking down prescribing to chapter and section level shows that the main contributor to the differences in prescribing levels stems from higher prescribing levels in chapter 4 (Central Nervous System medications) of both Analgesics and Antidepressants in Metropolitan practices. It is interesting that these higher levels are not seen in section 1 (Hypnotics and Anxiolytics) also as these are often co-prescribed. It is also interesting that there is no significant difference in the prescribing of drugs used in substance abuse between the two clusters. One possible explanation is that since there are two main centres for drug abuse in Northern Ireland, one in each cluster, this may be obscuring any differences. Examining the practices in relation to the level of deprivation seen in the area where they are located does show that a high percentage (72.2%) of Metropolitan practices are located in areas with high deprivation. Given these proportions, it is likely the deprivation levels attributed to the areas in which practices are located is one of the factors contributing to the differing prescription levels seen. Other factors such as patient demographics (age structure of the populations, ethnic and cultural differences in population composition etc.), in practitioner demographics (including age, gender, part-time/full-time status etc.), and in patient-full-time equivalent GP ratios (and consultation times) may also contribute^31,32^.

### Policy and practice implications

This paper provides a taxonomy for GP practice types that could be used on dashboards for comparing or benchmarking different practices allowing the possibility of applying standardisation to prescribing practices. These dashboards could be used by government or health authorities. In addition, the provision of a general archetype will allow for the identification of anomalous behaviours indicating the possibility of lack of services, overloading of services or fraudulent behaviour within specific geographical areas or practices. Analysis of specific medications will provide a clearer picture of the local population’s health highlighting the geographic location of high-risk areas for specific illnesses and providing a steppingstone for further research into the reasons for higher levels of illness observed in these locations.

### Limitations

This study has sought to categorise and track GP practices operating in Northern Ireland over time. The dataset on which categorisation was performed only became available from April 2018 limiting the available data to a 1-year period (April 2018–March 2019). In addition, only GP practices that operated during the whole period were included (333 practices) with no provision made for practices which closed or those opening during the period. Similarly, whilst prescription data is available from 2013, it was only possible to track the prescriptions associated with those categorised from July 2015 until December 2019. Any analysis prior to July 2015 would have to consider openings and closures for which no data is currently available. The analysis is based on tracking the location of the GP Practice issuing a prescription to the location of the pharmacy dispensing it. Whilst this study uses Number of Items per Registered patient as a proxy for the levels of sickness experienced, this may not be accurate as some GPs may be over prescribing or prescribing where anther GP would ask the patient to buy over the counter (e.g. paracetamol). It is likely that the majority of Registered Patients do not reside in the same Super Output Area as the practice they attend. As this does not necessarily reflect the actual residential location of the patient receiving the prescription, it is assumed that patients will dispense their prescriptions at their local pharmacy meaning that distance travelled can be used as a proxy. Similarly, the Population Density used as a feature in clustering practices is the Population Density of the Super Output Area in which the Practice is located. The deprivation ranking was assigned to each practice for the area in which they are located. Given that we have established that patients most likely travel to their practice and dispense their prescriptions closer to home, further research examining the deprivation of the areas covered by each practice is warranted.

## Conclusion

This paper has set out to investigate the relationship between the geographic location of GP practices (to categorise practices based on their geographic location) and their prescription profile. To do this we have linked each practice with their associated dispensing pharmacies in Northern Ireland to ascertain whether this has any effect on prescribing patterns. In doing so, we have presented a methodology to compute archetypes based on areas of interest (in this case geolocation attributes) for subsequent comparisons to determine if the archetypes differ in behaviour. It was found that it was possible to classify GP practices based on geolocation attributes and two different archetypes of GP practice were identified: Metropolitan and Non-Metropolitan (the labels Urban and Rural were not appropriate and no evidence was found for the Semi-Rural category commonly used by healthcare researchers in Northern Ireland). Average dispersion distances were calculated for each set of pharmacies dispensing prescriptions for each practice, the results supporting the two categorisations. Prescribing patterns were largely similar for each type with levels of prescribing higher in approximately half of the BNF chapters for the Metropolitan cluster. It was found that BNF chapter 4 (Central Nervous System) accounted for the largest proportion of variation between the identified clusters with sections 7 (Analgesics) and 3 (Antidepressant Drugs) being the main contributors.

The level of deprivation experienced in the local GP practice area was examined to see if this was a contributing factor to the observed prescribing levels. This analysis showed that it was likely that deprivation was a contributing factor although may not totally explain the differences seen.

Further study is required to analyse deprivation as an effect of prescribing behaviours and the effect the size of practice (in terms of number of doctors within the practice) have on prescribing behaviours with further work to interpret the differences in prescribing behaviours.

## Figures and Tables

**Figure 9:**
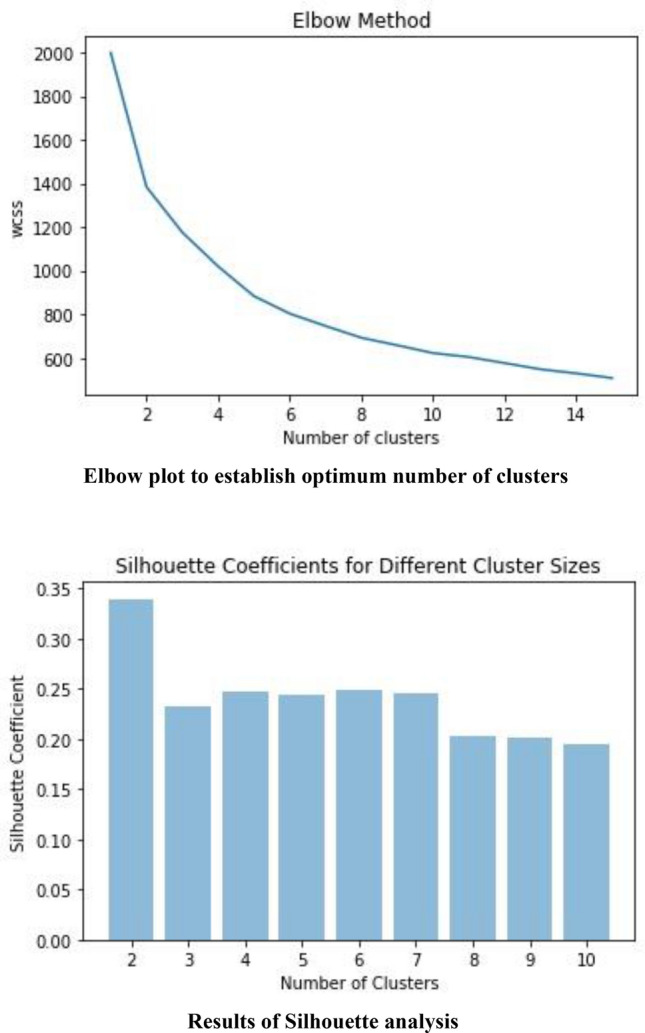
Results of Elbow Plot and Silhouette Analysis.

**Figure 10:**
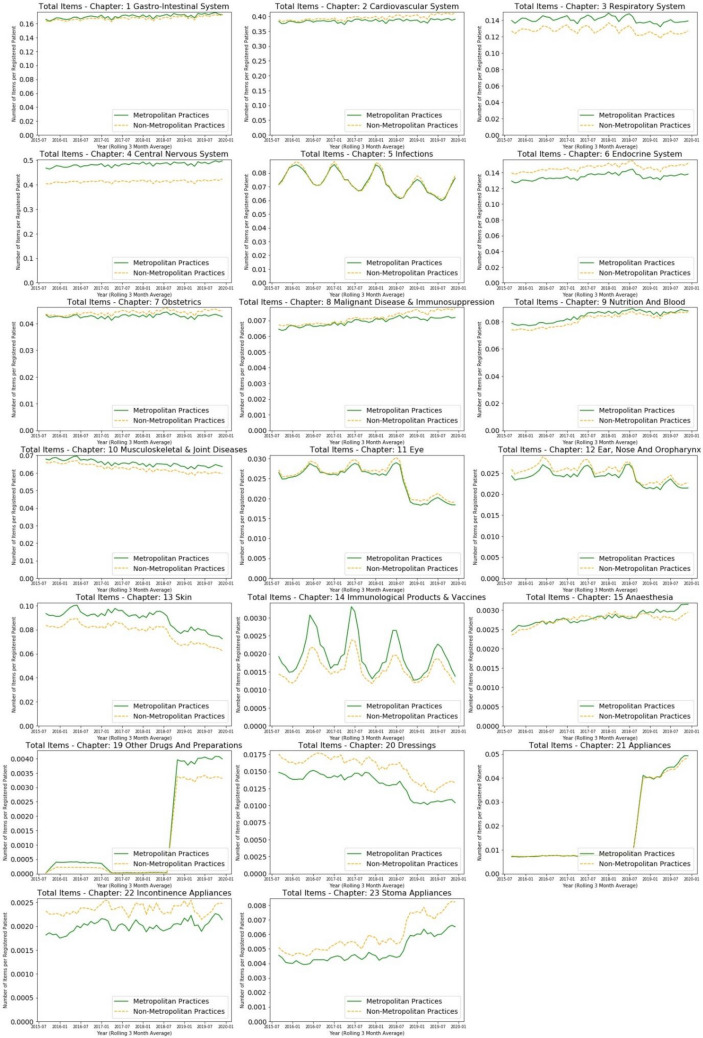
Total Items prescribed by cluster by BNF chapter.

## Data Availability

All data used in this study are publicly available.

## Appendix 1

See Fig. 9.

## Appendix 2

See Fig. 10.
